# Perioperative management of a pregnant patient with a mechanical heart valve undergoing appendectomy: a case report

**DOI:** 10.1097/MS9.0000000000002994

**Published:** 2025-02-07

**Authors:** Ibrahim Nagmeldin Hassan, Siddig Yaqub, Mohamed Ibrahim

**Affiliations:** a Faculty of Medicine, University of Khartoum, Khartoum, Sudan; b Faculty of Medicine, Karary University, Khartoum, Sudan; c Faculty of Medicine, Al-Neelain University, Khartoum, Sudan

**Keywords:** anticoagulation, appendectomy, low-molecular-weight heparin, mechanical heart valve, perioperative management, pregnancy

## Abstract

**Introduction and importance:**

Managing pregnant patients with mechanical heart valves who require non-cardiac surgery presents unique challenges, particularly in balancing anticoagulation to prevent thromboembolic complications while minimizing bleeding risks. This case report discusses the perioperative management of a pregnant patient with a mechanical mitral valve undergoing emergency appendectomy.

**Case presentation:**

A 28-year-old pregnant woman at 22 weeks gestation with a history of rheumatic heart disease and a mechanical mitral valve presented with acute appendicitis. Anticoagulation therapy was shifted from warfarin to low-molecular-weight heparin (LMWH) to mitigate the risks of bleeding and teratogenicity. Intravenous unfractionated heparin (UFH) was used perioperatively to maintain adequate anticoagulation. A multidisciplinary team guided the patient’s care, and she underwent a successful laparoscopic appendectomy with stable maternal and fetal outcomes.

**Clinical discussion:**

Perioperative anticoagulation in pregnant women with mechanical heart valves is critical to avoiding valve thrombosis while minimizing the risk of bleeding. In this case, using LMWH as a bridging therapy and UFH during surgery proved effective in balancing these risks. The case underscores the importance of a coordinated multidisciplinary approach to optimize maternal and fetal outcomes during emergency surgery.

**Conclusion:**

This case highlights the complexities of managing anticoagulation in pregnant patients with mechanical valves requiring non-cardiac surgery. With appropriate anticoagulation adjustment, multidisciplinary planning, and vigilant perioperative monitoring, both maternal and fetal outcomes can be favorable in such high-risk situations.

## Introduction

Pregnancy in patients with mechanical heart valves presents substantial risks due to the need for continuous anticoagulation to prevent valve thrombosis. Managing such patients during non-cardiac surgery requires balancing the anticoagulation regimen to reduce both thrombotic and bleeding risks while minimizing harm to the fetus^[1]^. Appendectomy, being the most common non-obstetric emergency surgery during pregnancy, adds to the complexity in such cases^[2]^. This case report describes the successful perioperative management of a pregnant patient with a mechanical mitral valve undergoing emergency appendectomy.

**Highlights**

- Managing anticoagulation in pregnant patients with mechanical heart valves requires careful balancing to prevent valve thrombosis and minimize bleeding risks.
- Low-molecular-weight heparin (LMWH) and intravenous unfractionated heparin (UFH) are effective alternatives to warfarin in pregnant patients undergoing emergency surgery.
- A multidisciplinary approach is essential to ensure favorable maternal and fetal outcomes during high-risk perioperative scenarios.

## Case presentation

A 28-year-old woman, gravida 2 para 1, at 22 weeks gestation, presented to the emergency department with a 24-hour history of worsening right lower quadrant abdominal pain, associated with nausea and vomiting. The patient had a significant medical history of rheumatic heart disease, for which she had undergone mitral valve replacement 3 years prior with a mechanical prosthetic valve. Since her surgery, she had been on long-term anticoagulation therapy with warfarin, 5 mg daily, to maintain a therapeutic international normalized ratio (INR) of 2.5–3.5. Her medical history revealed no other medications apart from warfarin. At presentation, her vital signs included a blood pressure of 110/70 mmHg, a heart rate of 85 beats per minute, a respiratory rate of 20 breaths per minute, and an oxygen saturation of 98% on room air. Physical examination revealed tenderness in the right lower quadrant with guarding and rebound tenderness, suggesting acute appendicitis. Fetal heart rate was 140 beats per minute on Doppler examination, with no signs of fetal distress.

Given the patient’s pregnancy and her mechanical heart valve, anticoagulation management became the primary concern. Warfarin was immediately discontinued to reduce the risk of intraoperative bleeding rather than solely due to concerns of teratogenicity. Although teratogenic risks are minimized beyond the first trimester, perioperative bleeding risks necessitated the switch to low-molecular-weight heparin (LMWH)^[3]^, enoxaparin, at a therapeutic dose of 1 mg/kg subcutaneously twice daily. Her INR, upon admission, was 2.5. As an additional precaution, intravenous unfractionated heparin (UFH) was administered at a continuous infusion rate of 18 units/kg/h, with adjustments made to achieve an activated partial thromboplastin time (aPTT) of 60–80 s. While there is limited evidence supporting the simultaneous use of LMWH and UFH in such scenarios, this approach was deemed necessary due to the high thrombotic risk posed by the mechanical valve^[4]^. A multidisciplinary team consisting of cardiology, hematology, obstetrics, and anesthesia specialists was convened to discuss the optimal approach to perioperative management. The decision was made to perform an emergency laparoscopic appendectomy under general anesthesia. Preoperative echocardiography demonstrated normal left ventricular function with an ejection fraction of 60%, and Doppler studies confirmed good mechanical valve function. Fetal ultrasound showed a viable fetus with no abnormalities, and continuous fetal monitoring was planned for the perioperative and postoperative periods.

The patient was taken to the operating theater, where general anesthesia was induced with propofol 2 mg/kg, fentanyl 2 µg/kg, and rocuronium 0.6 mg/kg. Anesthesia was maintained with sevoflurane 1.5% and a remifentanil infusion at 0.1 µg/kg/min. Invasive hemodynamic monitoring was established using an arterial line and central venous catheter. The patient’s initial intraoperative vitals were stable, with a blood pressure of 105/65 mmHg, a heart rate of 90 beats per minute, and an oxygen saturation of 97%. Fluid management was guided by stroke volume variation and central venous pressure, which was maintained between 8 and 12 mmHg. Norepinephrine was administered at a starting dose of 0.03 µg/kg/min to maintain the mean arterial pressure within the range of 65–75 mmHg to ensure adequate perfusion without exacerbating bleeding risks. The surgical procedure proceeded without complications, and the appendix was successfully removed. Intraoperative blood loss was estimated at 150 mL. The total duration of surgery was 90 min, during which the patient’s hemodynamic status remained stable. There were no significant fluctuations in her vital signs, and no arrhythmias or signs of valve dysfunction were observed.

Postoperatively, the patient was transferred to the intensive care unit (ICU) for continuous monitoring. Fetal heart rate monitoring was initiated immediately after surgery, and the fetus remained stable throughout the postoperative period. Enoxaparin was resumed at 1 mg/kg twice daily 6 h after surgery to prevent valve thrombosis. Intravenous UFH was discontinued once the patient’s aPTT values were within the therapeutic range. Warfarin was reintroduced on postoperative day 3, with a target INR of 2.5–3.5. During the bridging period, INR was closely monitored, and LMWH was continued until therapeutic warfarin levels were achieved. The patient’s postoperative vitals remained stable, with a blood pressure of 110/70 mmHg, a heart rate of 80 beats per minute, and an oxygen saturation of 96%. She experienced no complications related to either anticoagulation or the surgical procedure. On postoperative day 5, the patient was discharged from ICU to the obstetrics ward for further observation. Fetal ultrasound was performed before discharge and showed normal fetal growth and development. The patient was discharged home on postoperative day 7 with warfarin therapy and a scheduled follow-up with cardiology and obstetrics. Throughout her recovery, she remained symptom-free, and both she and her fetus progressed without further complications.

## Discussion

The perioperative management of pregnant patients with mechanical heart valves undergoing non-cardiac surgery is a complex and high-risk endeavor, requiring meticulous balancing of anticoagulation and hemodynamic stability. In this case, the patient had a mechanical mitral valve and required emergency surgery for acute appendicitis. The challenges included preventing thromboembolism, managing the risks of bleeding, and ensuring fetal well-being throughout the perioperative period^[1]^.

Pregnant women with mechanical heart valves are at significant risk of thromboembolic complications, particularly during periods of interrupted anticoagulation^[2]^. Warfarin, the preferred anticoagulant for patients with mechanical valves, poses teratogenic risks, especially during the first trimester. In this case, as the patient was in her second trimester, warfarin had been continued until the need for surgery arose. Upon diagnosis of appendicitis, warfarin was immediately discontinued to reduce the risk of intraoperative bleeding and was replaced with low-molecular-weight heparin (LMWH), which has a better safety profile during pregnancy^[3]^.

LMWH was used as bridging therapy due to its efficacy in preventing valve thrombosis without the teratogenic effects associated with warfarin. Additionally, intravenous unfractionated heparin (UFH) was administered perioperatively to ensure adequate anticoagulation in the critical hours leading up to surgery, with dosage adjusted based on aPTT values. This strategy allowed for rapid modulation of anticoagulation around the time of surgery, balancing the competing risks of valve thrombosis and bleeding^[5]^.

The multidisciplinary approach taken in this case, involving cardiology, obstetrics, anesthesia, and surgery teams, was essential in optimizing maternal and fetal outcomes. Close collaboration between specialists ensured that both anticoagulation management and fetal monitoring were integrated into the perioperative plan. Anesthesia choices also played a crucial role, as the team selected a general anesthetic regimen that minimized hemodynamic fluctuations while maintaining fetal oxygenation. Invasive hemodynamic monitoring allowed for real-time assessment of the patient’s cardiovascular status, which was critical given the increased demands on cardiac output during pregnancy^[6]^.

Postoperative care focused on restoring anticoagulation with warfarin while minimizing the risk of surgical bleeding. Bridging with LMWH was continued until the INR reached therapeutic levels, and fetal monitoring remained a priority throughout the postoperative period. This case emphasizes the importance of careful anticoagulation adjustment and the need for vigilant postoperative monitoring in pregnant patients with mechanical valves undergoing non-cardiac surgery. This case report was written in accordance with the SCARE 2023 criteria (Surgical CAse REport guidelines) to ensure proper structure, reporting standards, and quality for surgical case reports^[7]^.

## Conclusion and recommendations

The management of a pregnant patient with a mechanical heart valve undergoing emergency appendectomy highlights the complex interplay between anticoagulation, maternal safety, and fetal well-being. This case demonstrates the importance of an individualized, multidisciplinary approach to perioperative care. Transitioning from warfarin to LMWH and using intravenous UFH for bridging during surgery allowed for safe anticoagulation while minimizing the risks of bleeding and valve thrombosis. The limitations of case presentations, such as the inability to generalize findings, are acknowledged. However, this case provides valuable insights into the successful management of similar high-risk situations. The successful outcome in this case reinforces the need for careful preoperative planning, intraoperative monitoring, and postoperative management in such high-risk patients. A coordinated effort between surgical, obstetric, cardiology, and anesthesia teams is vital to optimize both maternal and fetal outcomes in these challenging cases.

## Data Availability

The dataset used and/or analyzed during the study is available from the corresponding author upon reasonable request.

## References

[1] D’Souza R Ostro J Shah PS. Anticoagulation for pregnant women with mechanical heart valves: a systematic review and meta-analysis. Eur Heart J 2017;38:1509–16. 28329059 10.1093/eurheartj/ehx032 PMC5429939

[2] Franca Neto AH Amorim MM Nóbrega BM. Acute appendicitis in pregnancy: literature review. Rev Assoc Med Bras (1992) 2015;61:170–77. 26107368 10.1590/1806-9282.61.02.170

[3] Basude S Hein C Curtis SL. Low-molecular-weight heparin or warfarin for anticoagulation in pregnant women with mechanical heart valves: what are the risks? A retrospective observational study. BJOG 2012;119:1008–13. 22568528 10.1111/j.1471-0528.2012.03359.x

[4] Daughety MM Zilberman-Rudenko J Shatzel JJ. Management of anticoagulation in pregnant women with mechanical heart valves. Obstet Gynecol Surv 2020;75:190–98. 32232497 10.1097/OGX.0000000000000751 PMC9299952

[5] Seshadri N Goldhaber SZ Elkayam U. The clinical challenge of bridging anticoagulation with low-molecular-weight heparin in patients with mechanical prosthetic heart valves: an evidence-based comparative review focusing on anticoagulation options in pregnant and nonpregnant patients. Am Heart J 2005;150:27–34. 16084147 10.1016/j.ahj.2004.11.018

[6] Wallenburg HC. Invasive hemodynamic monitoring in pregnancy. Eur J Obstet Gynecol Reprod Biol 1991;42:S45–51. 1809608

[7] Sohrabi C Mathew G Maria N. The SCARE 2023 guideline: updating consensus Surgical CAse REport (SCARE) guidelines. Int J Surg Lond Engl 2023;109:1136. 10.1097/JS9.0000000000000373 PMC10389401 37013953

